# BDNF Genetic Variant and Its Genotypic Fluctuation in Major Depressive Disorder

**DOI:** 10.1155/2021/7117613

**Published:** 2021-11-01

**Authors:** Caroline Ferreira Fratelli, Jhon Willatan Siqueira, Bruna Rodrigues Gontijo, Maurício de Lima Santos, Calliandra Maria de Souza Silva, Izabel Cristina Rodrigues da Silva

**Affiliations:** ^1^Health Sciences and Technologies Program, University of Brasilia, Faculty of Ceilândia, Brasilia, DF, Brazil; ^2^University of Brasilia, Faculty of Ceilândia, Brasilia, DF, Brazil

## Abstract

Major depressive disorder (MDD) still has an unknown etiology and mechanisms. Many studies have been conducted seeking to associate and understand the connection of different genetic variants to this disease. Researchers have extensively studied the brain-derived neurotrophic factor (BDNF) Val66Met genetic variant in MDD; yet, their findings remain inconsistent. This systematic review sought to verify the GG (Val/Val) genotype frequency fluctuation in different populations with MDD. For this, we searched in different databases and, after applying the eligibility criteria, selected 17 articles. Most studies demonstrate the higher frequency of the ancestral (wild) GG (Val/Val) genotype, although associations of the polymorphic A (Met) allele, changes in BDNF protein serum levels, or both were also found in MDD, whether related to the disease's development or other factors. Nevertheless, despite these findings, disagreements between several studies are seen. For this reason, further BDNF Val66Met genetic variant studies should not only bridge the gap in the knowledge of this polymorphism's role in MDD's different facets but also analyze the genotypic and phenotypic heterogeneity in different populations to help provide a better quality of life for patients.

## 1. Introduction

Depression is considered a mental disorder with high disability globally [1]. With different episodes, lasting at least two weeks, changes in emotions, such as sadness, irritation, or emptiness, and dysfunctions in cognition and neurovegetative functions, which affect the individual's ability to function, major depressive disorder (MDD) is one type of depressive disorders [2]. MDD causes significant public health concerns as it is the most prevalent, underdiagnosed, and undertreated mental disorder, thus requiring an expansion of screening methods [1, 3].

According to the WHO (World Health Organization) [1], MDD cases increased by 18.4% between 2005 and 2015, although this increase might also be due to population growth. The risk for MDD development involves the performance of several genes and associations of diverse environmental factors, and, even with the advances in MDD neurobiology, no mechanism has yet explained all facets of this disease or its specific etiology [4]. Compared to men, women tend to be more susceptible to MDD [4]. Its prevalence rate also varies with age increasing from 1-2% at 13 [5] and 3-7% (15-19 years) [5] to almost 5% (60-69 years) in males and 8% (60-69 years) in females [4, 6], with adults grouped between 55 and 74 of age having a higher peak than other ages [4, 6]. Interestingly, the male/female ratio vary from equal during childhood [5] to 1 : 2 during adolescence [5] to 5 : 8 in older adults [4, 6]. In other words, depression is not specific to a particular age group and can affect children and adolescents.

The brain-derived neurotrophic factor (BDNF) protein belongs to the neuronal growth factor family and is detected, besides the neuronal tissues, in nonneuronal tissues, such as endothelial cells, cardiac cells, vascular smooth muscle, leukocytes, megakaryocytes, and platelets [7]. Located on the human 11p14.1 chromosome, the *BDNF* gene carries the SNP rs6265, which substitutes a valine (G) with a methionine (A) at codon 66 (Val66Met) of the BDNF (pro-BDNF) precursor [4, 8], generating a decrease in the BDNF protein secretion [9–11]. Hence, allele A (met) presence might be related to a lower BDNF activity-dependent secretion [10].

Studies assessing *BDNF* gene expression and peripheral levels are varied, especially when related to MDD. The findings are incongruous, presenting a significant association in some studies and none in others [12], probably due to the genotypic and phenotypic heterogeneity in different populations analyzed. Hence, to understand the effect of the *BDNF* Val66Met (rs6265) gene variant on MDD's different facets, this systematic review verified the GG (Val/Val) genotypic frequency fluctuations and associations to MDD, in several populations, through original articles published between 2016 and 2020.

## 2. Material and Methods

### 2.1. Search Strategy and Selection Criteria

This systematic review followed the guidelines established by Prisma proper for systematic reviews and meta-analyses and is registered in the Prospective Register of Systematic Reviews (PROSPERO) under CRD42020218671.

The inclusion criteria were based on the aspects of Population, Exposure, Comparison, Outcome, and Study type (PECOS), in which (1) population: research participants with major depressive disorder (MDD); (2) exposure: BDNF Val66Met (rs6265) genetic variant; (3) comparison: the dominant ancestral GG (Val/Val) genotypic frequency; (4) outcome: the dominant ancestral GG (Val/Val) genotypic frequency fluctuation in different populations; (5) study type: observational and intervention.

For this, open access observational or interventional studies that described the BDNF Val66Met (rs6265) genetic variant genotypic frequencies in MDD research participants and presented laboratory methods were accepted, according to the eligibility criteria. However, studies with incomplete data, including statistical data, reviews, meta-analyses, and abstracts, were excluded.

The research was conducted on November 30, 2020, employing the Web of Science, PubMed, and Virtual Health Library (VHL) databases. Although there were no language restrictions, a filter was applied to select complete texts and articles published in the last four years (2016 to 2020). Indexed terms (descriptors) researched reflected the exposure and the outcome of interest for this review and were suitable for the Medical Subject Headings (MeSH) vocabulary thesaurus. These descriptors were “polymorphism genetic,” BDNF, and “Major depressive disorder,” combined by the Boolean operator “AND.”

### 2.2. Study Selection and Data Extraction

Two reviewers (CF and JS) performed the article selection in two phases. In the first phase, each reviewer independently analyzed each article's title and abstract, checking the eligibility according to the PECOS strategy. For this phase, the Rayyan tool, developed by the Qatar Computing Research Institute (QCRI) [13], was used to assist in this initial analysis and remove all duplicates. In the second phase, the same two reviewers (CF and JS) independently analyzed the full text of the articles that passed the first phase for preestablished eligibility criteria. For this, Mendeley Desktop version 1.19.4 software was used.

In both phases, the reviewers discussed any disagreements or doubts, and if the issue was not resolved, a third reviewer (IS) was consulted. Predefined data were then extracted from the selected articles, independently by the two reviewers (CF and JS), to a spreadsheet in Microsoft Office Excel: author, study title, objective, year of publication, the country in which study was performed, the studied genetic variants, the dominant ancestral GG (Val/Val) genotypic frequency, sample size, laboratory methodology, main result, and *p* value. Any doubt, the corresponding author (IS) was contacted to solve the question.

### 2.3. Bias Risk in Each Study

Risk models can be based on two forms: evaluating only genetic variants or analyzing genetic and environmental risk factors [14, 15]. The selected studies' bias risk was analyzed by applying the Genetic RIsk Prediction Studies (GRIPS) guideline to determine their quality [14]. From a total of 25 GRIPS items, 20 items were contemplated when assessing the selected studies' quality, with each term evaluated for their presence or absence. The articles were considered of good quality if they presented at least 75% of the items initially described.

Two reviewers (CF and JS) independently analyzed each selected article by the GRIPS guideline, and any disagreements found were resolved after a discussion with the third reviewer (IS).

## 3. Results

### 3.1. General Characteristics of the Studies

**Figure 1:**
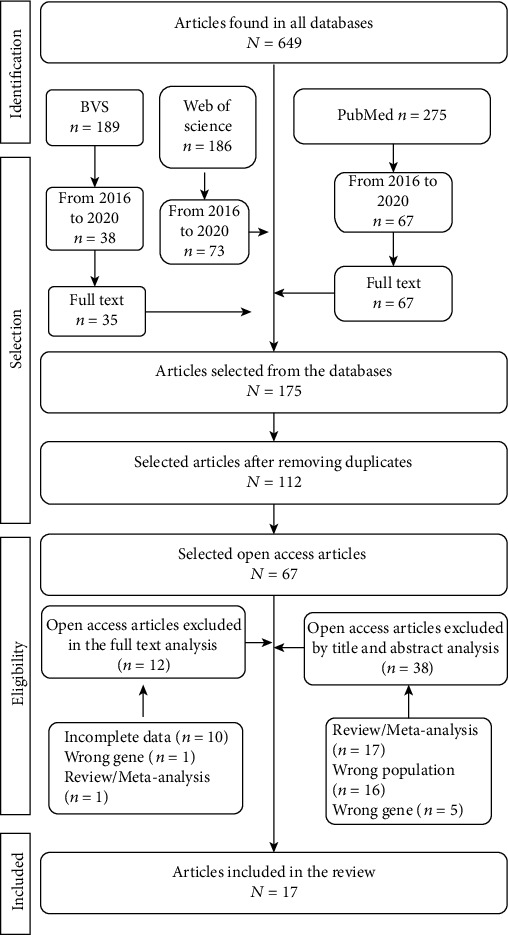
Performed bibliographic research flowchart.

**Figure 2:**
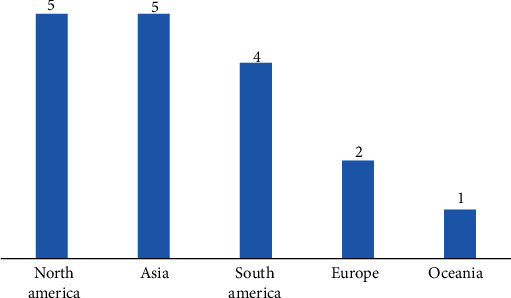
Number of articles per continent.

In summary, we initially identified a total of 650 scientific articles. After removing duplicates and checking for open access articles, 67 titles and abstracts were selected for analysis following the aspects delimited in the PECOS strategy. The application of the preestablished inclusion and exclusion criteria rendered 17 articles analyzed in this systematic review (Figure 1). Most of these studies were conducted on North America (Canada and the United States of America) and Asia (China, Malaysia, and the Republic of Korea) continents. Nonetheless, we also found studies in South America, Oceania, and Europe (Figure 2).  Table 1 presents the information extracted from the selected scientific articles.

Analyzing the articles' study population, all had a higher frequency of females, except for the study by Youssef et al. [16] composed of 10 women and 35 depressed men. Tatham et al. [17], on the other hand, did not show the sex variable frequency in their sample. Regarding the age variable, all participants from all studies were over 18 years of age.

Table S1 describes the rejected articles that did not fit the PECOS strategy and the eligibility criteria (see supplementary material).

### 3.2. BDNF GG (Val/Val) Genotype Frequency in Different Populations with MDD

The BDNF Val66Met (rs6265) genetic variant has been evaluated in several populations, such as Americans [16], Brazilians [26], Argentineans [25], and Malaysians [20]. In these studies, the polymorphic A (Met) allele seems to be somehow related to MDD, either by reducing [16] or raising [21, 26] BDNF protein levels or even by increasing the risk [16, 20, 25] or protection against [23] disease development. Namely, no conformity is present in the literature regarding the rs6265 polymorphism presence and the MDD occurrence.

**Figure 3:**
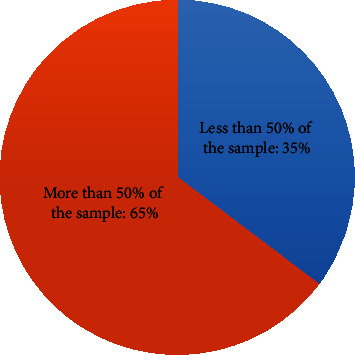
GG (Val/Val) genotype frequency distribution of the BDNF (rs6265) genetic variant in MDD sample population from 2016 to 2020.

When observing only the BDNF Val66Met (rs6265) genetic variant frequency and genotypic distribution (Table 1), the GG (Val/Val) genotype was frequent in more than 50% of the MDD sample studied in 65% of the analyzed studies [17, 18, 19, 21, 23, 25, 26, 30–33] (Figure 3). Its frequency is lower in only few studies [16, 20, 22, 27–29].

## 4. Discussion

### 4.1. BDNF (rs6265) Genetic Variant and Its Genotypic Frequency in MDD

MDD is considered a complex, multifactorial disease and unknown etiology that requires an association of environmental and genetic factors for its development. BDNF protein, widely present in the Central Nervous System, contributes to neurons' growth, survival, differentiation, and plasticity by maintaining an association with tropomyosin receptor (TrkB) [34]. Despite its importance in the nervous system and association with reduced gene function and protein concentration, the literature presents no conformity regarding the A (Met) allele of the rs6265 polymorphism role in MDD occurrence, a fact that might have ethnic heterogeneity as one of the possible explanations.

Aldoghachi et al. [20] verified the association of three BDNF genetic variants (rs6265, rs1048218, and rs1048220) in 300 depressed Malaysian participants. When analyzing only the rs6265 variant, 73 MDD participants had the dominant ancestral GG (Val/Val) genotype, 100 MDD participants had the recessive polymorphic AA (Met/Met) genotype, and 127 had the heterozygous GA (Val/Met) genotype. The logistic regression demonstrated that two copies of the recessive polymorphic allele (A) were necessary to increase the risk of developing MDD by 1.71 times (*p* = 0.0035; OR = 1.75; 95%CI = 1.19‐2.45) [20].

Ribeiro et al. [35] found a similar result in a Caucasian population: the participants with the polymorphic AA (Met/Met) genotype had a greater chance of developing MDD compared to the other genotypes (*p* = 0.005; OR = 1.7; 95%CI = 1.17‐2.47). In a Taiwan study, the AA (Met/Met) genotype carriers were 2.49 times more likely to develop MDD (*p* = 0.001; OR = 2.49; 95%CI = 1.40‐4.46) [36]. These findings point to BDNF as a potent biomarker for early MDD screening in the near future [20].

Bassi et al. [25] also determined the polymorphic allele's risk ratio with MDD. In their study with 95 depressed Argentineans, most participants had the G allele (Val) (78%) of the rs6265 variant. Participants with at least one copy of allele A (Met), along with specific alleles (L/S; 10/12; T/C; and 3/3) of other possible MDD-related genes (5HTTLPR, 5HTTVNTR, HTR2A, and APOE), had an increased risk of developing depression (*p* = 0.004; OR = 5.99; 95%CI = 1.66‐21.56) [25].

In the Chinese population, Sun et al. [37] also investigated a possible interaction between the *5HTTLPR* and *BDNF* (rs6265) genes with the risk of developing MDD and found no significant association between MDD and BDNF, solely with the 5HTTLPR genetic variant, when examined individually. However, when analyzed together, 5HTTLPR and BDNF (rs6265) genotypes showed a significant interaction related to MDD [37]. To confirm this association between the 5HTTLPR heterozygous (LS) and homozygous (LL/SS) genotype with the BDNF dominant ancestral GG (Val/Val) genotype, 459 Chinese MDD participants were analyzed, and the combination of the LS (5HTTLPR) and GG (BDNF) genotypes increased three times the risk of developing MDD [37].

In this same perspective of associating different genetic variants in MDD, Kostic et al. [32] evaluated the accumulation effect of the functional BDNF, COMT, and SERT polymorphisms in 85 Serbian MDD patients' symptom severity. Most of the MDD sample (69%) had the BDNF (rs6265) dominant ancestral GG (Val/Val) genotype and showed a significant difference to the group considered healthy (*p* = 0.01) [32]. Notably, the highest probability of finding a BDNF dominant ancestral GG (Val/Val) genotype carrier that also carried the Met allele (COMT) and the L allele (SERT) was likewise in the MDD patients' group. Hence, at least one relative in the MDD group will probably be affected by MDD (*p* = 0.02). This higher probability of a relative being affected by MDD also occurs when at least two of these variants were evaluated (*p* = 0.04) [32]. In other words, as the number of the genetic variants' associations increased, it increases the risk of developing depressive disorder and its severity.

Endeavoring to determine the contribution of APOE, HSPA1A, SLC6A4, BDNF, and HTR2A genetic variants to MDD development, Kitzlerová et al. [23] performed a case-control study with 68 MDD inhabitants of the Czech Republic, in which 53 had the GG (Val/Val) genotype (77.9%), 11 the AA (Met/Met) genotype (16.6%), and 4 the GA (Val/Met) genotype (5.9%). The interactions between gene polymorphism HSPA1A (rs1008438), SLC6A4 (rs4795541), and BDNF (rs6265), or HSPA1A (rs1043618 and rs1008438), APOE (rs429358), and BDNF (rs6265) demonstrated a significant accumulation effect when associated with MDD (*p* = 0.016), supporting the idea that MDD may be associated with the interaction of multiple genetic pathways [23], as was seen in other studies [25, 32]. In other words, a synergistic effect of these genes may be influencing inflammatory, serotonergic, and neurotrophic pathways [23].

In general, the results present no conformity regarding the role of the polymorphic A (Met) allele presence in MDD. Ethnic heterogeneity may explain the G (Val) allele frequency fluctuation, as shown in Table 1. However, the sample size might also be an influencing factor in genotypic studies.

Shen et al. [38] verified this same nonconformity when analyzing 1064 healthy individuals from 57 populations worldwide. The polymorphic A (Met) allele frequency increased from 0.55% to 19.9% when comparing individuals from sub-Saharan Africa and Europe. The same occurred when compared with individuals from Asia (72%) [38]. Therefore, the A (Met) allele frequency varied from 0.55% to 72%, depending on the population studied.

In addition to ethnic heterogeneity, sample size, age, and gender effects, environmental factors also brought controversies and poorly resolved issues that hinder study replication [7, 38]. For this reason, several studies propose that future research should analyze this association in different populations controlling these constituents to understand the actual connection between genetics and MDD [20, 37, 39].

### 4.2. BDNF Genetic Variant and the Central Nervous System

Depression involves not only changes in monoamines, such as serotonin, availability but also brain structure abnormalities. These structure and function changes are in brain regions related to emotion: the prefrontal cortex, cingulate cortex, hippocampus, and tonsil [40, 41]. As fractional anisotropy (FA) measures neuronal cells' integrity, FA values' reductions indicate these cells' loss [42]. Hence, several studies often analyzed genetic and environmental factors' effect on these values and their association with MDD [17, 28, 43].

BDNF (rs6265) polymorphism interactions with MDD seem to affect the uncinate fasciculus (UF) region in the brain [17, 28, 43]. FA values decreased significantly in MDD patients with at least one allele A (Met) compared to dominant ancestral GG (Val/Val) genotype carriers [28, 43]. Conversely, in the healthy group's research participants, FA values were significantly higher in A (Met) allele carriers than the GG (Val/Val) genotype carriers [28, 43]. The BDNF polymorphism moderated the correlation between depression severity and the FA values in UF (*p* = 0.02) [17]. That is, as the depression severity increased, the A (Met) allele effects on the UF region became more evident by their carriers' reduced FA values compared to those with the G (Val) allele.

Another study by Tatham et al. [30] also found a significant effect of the BDNF (rs6265) genetic variant on the FA values in left UF after antidepressant use (*p* = 0.009). MDD participants with high FA values in the left UF improved their depression severity after using antidepressants, with GG (Val/Val) genotype carriers having higher FA values than A (Met) carriers. Hence, the genetic factor effect in limbic neural structure, i.e., the neuronal connectivity, may indirectly affect the response to antidepressants [30].

However, not only the genetic factors produce disease. The psychosocial environment, including childhood adversities and abuse, also does. Consistently, when examining solely environmental factors, healthy participants with childhood adversities exhibit significantly reduced FA values in UF compared with other participants [17, 44–46]. In comparison, MDD patients that suffered childhood adversities presented increased FA values in different brain regions [17, 45]. These psychosocial environmental factors possibly interact with the BDNF Val66Met polymorphism affecting the neural structure [17, 28]. Unfortunately, one of Han et al. [28] study's limitation was not correlating their FA value results with environmental changes during their research.

Jaworska et al. [31] found no effect of the BNDF (rs6265) polymorphism on cortical or other regions' thickness nor in the hippocampus volume; the latter result was similar to few other studies [47, 48]. In comparison, Cao et al. [33] detected a reduction in the hippocampus volume in patients with the polymorphic A (met) allele, agreeing with most of the evidence [17, 28, 30, 33, 43]. Jaworska et al. [31] justify this discrepancy with their small sample size, composed of 58 participants (MMD = 43; control = 15).

Therefore, despite the controversies, BDNF plays a role in the structure of different brain regions, and to this end, future studies should strive to overcome the gaps that remain about its function in the Central Nervous System.

### 4.3. BDNF Genetic Variant in Suicide and Childhood Adversity

Childhood adversities' connection to suicide and mental disorders is not well understood. Mistreatment in childhood usually associates with a depressive disorder, which often is a cause of the high suicide rate in old age [49–51]. Childhood adversity also correlates prominently with a family history of mental disorders in groups with suicide attempts [49–51].

Youssef et al. [16] analyzed the BDNF interrelation with suicide, MDD, and reported childhood adversities in an unidentified postmortem population, divided into two groups of suicidal (37) and nonsuicidal (53), and then regrouped them into depressed (45) and nondepressed (45) participants. No statistical difference was determined between the distinct groups when the “childhood adversities” variable was analyzed (*p* = 0.658); yet, a statistically significant difference was determined regarding the “death by suicide” variable (*p* < 0.001) [16].

Similarly, a study conducted with only Asian and Korean participants found that the “suicide attempts” variable was more present in patients with at least one G (Val) allele of the BDNF gene (rs6265) than in AA (Met/Met) genotype patients (*p* = 0.015) [16]. Corresponding results were seen in a study in France and Switzerland [52]. In Brazil, Schenkel et al. [53] related “suicide attempts” to the A (Met) allele rather than the G (Val) allele. Interestingly, Korean participants presented mixed results [54–57], demonstrating a lack of consensus on this association. Although no significant association between the BDNF genetic variant and the “attempted suicide” variable in a Chinese study (*p* = 0.807), Brunoni et al. [19] found low BDNF protein serum levels might be related to attempted suicide [22].

Observing the BDNF protein levels in brain regions, Youssef et al. [16] noted a decrease in its serum levels associated with the variables “childhood adversities” and “death by suicide.” However, the BDNF genetic variant did not differ between suicidal and nonsuicidal decisions (*p* = 0.24) or reported childhood adversities (*p* = 0.62) [16]. Similarly, Chiou and Huang [58] reported that the BDNF protein serum levels were lower in depressed patients who attempted suicide (*p* = 0.038) [58]. In a cohort study with 84 participants who attempted suicide, Eisen et al. [59] found no relationship between BDNF serum levels and the “attempted suicide” variable (*p* = 0.82), though the study should be replicated in a larger sample population.

An unregulated stress response system may explain the association between low BDNF protein levels, suicide, and childhood adversity. The mistreatment suffered throughout life might cause reduced BDNF transcript levels (gene expression) through its gene methylation in the prefrontal cortex in adults, in addition to stress, which are possible chronic effects that accrue from childhood adversity. In suicidal behavior, stress may be considered an acute effect. Therefore, these events' influence may alter the BDNF gene's translation modifying its protein levels [16, 60, 61].

The BDNF gene and its protein levels may be affected by life's adversities and suicide attempts [16, 53, 57], proving that environmental and genetic factors go together for MDD development. Regardless, additional studies in different populations are necessary to reduce the controversies regarding these variables' association [54, 56, 57, 62].

### 4.4. BDNF Genetic Variant and Treatment Response

Little is understood about the relationship between BDNF and antidepressant response [58, 63]. Over time, many MDD pathophysiology mechanisms have been investigated, relating it to dopaminergic, noradrenergic, glutamatergic, and serotonergic systems, in addition to changes in inflammation markers [7, 26]. What is known is that BDNF acts as a transducer, i.e., a communication link between the antidepressant drug and the neuronal alterations that result in symptom improvements [64]. Depressed patients receiving appropriate treatment significantly increase BDNF protein serum levels, leading to BDNF being perceived as a biomarker of drug treatment response, especially with Selective Serotonin Reuptake Inhibitors (SSRIs), for its significant role in the serotonergic system [22], though there are still controversies [27, 58].

SSRIs are generally the first choice in antidepressant treatment, presenting fewer adverse effects than other antidepressant classes [65–67]. Caldieraro et al. [26] analyzed the pharmacotherapy used in participants separated according to the BDNF (rs6265) gene's genotype. Ancestral GG (Val/Val) genotype participants (60%) had significantly higher rates of SSRIs use compared with the group with at least one polymorphic A (Met) allele present (88.9%) (*p* = 0.024) [26].

Antidepressant use might affect the BDNF protein serum levels [19, 20]. Froud et al. [21] found that the recent use of antidepressants in the last 12 months was a substantial factor for changes in the BDNF protein serum levels. Chiou and Huang [58] noted the BDNF plasma protein levels were lower in MDD patients taking antidepressants for the first time than the group considered healthy, which might have led to the assumption of BDNF having a fundamental role in the serotonergic system that causes neurobiological and clinical changes. A meta-analysis confirmed this assumption that, after using SSRIs, the BDNF protein expression is increased [63]. However, Chiou and Huang [58] also found BDNF serum levels unaltered (*p* = 0.113) after six years of treatment with antidepressants. Kao et al. [68] similarly confirmed the lack of association between the BDNF gene rs6265 functional polymorphism and SSRI therapy response. This difference might imply that the antidepressant effect on the BDNF protein serum levels is time-dependent, indicating a need to regard SSRI therapy duration as a variable to compare studies.

Hennings et al. [69] crossed with three BDNF gene variants (rs2049046, rs11030094, and rs6265) to the different therapeutic classes, finding a significant association between the rs2029046 variant and noradrenaline reuptake inhibitors (*p* = 0.04), between the rs11030094 variant and the tricyclic antidepressant class (*p* = 0.02), and none between the rs6265 variant and different therapeutic classes evaluated. They concluded that the rs2049046 and rs11030094 genetic variants, associated with the antidepressant response, impact the Hypothalamus-Pituitary-Adrenal Axis (HPA) regulation in MMD. These findings were entirely novel for these two regions [69].

A case-control study divided the 68 MMD Czech Republic participants into two groups: “responders to treatment” and “nonresponders to treatment”—SSRIs or other antidepressant classes (mitarzapine, velanfaxine, and trazodone) [23]. BDNF rs6265 heterozygous GA (Val/Met) genotype showed a lower frequency in the “responders to treatment” group (14.0%) compared to the “nonresponders to treatment” group (23.5%). No statistically significant *p* value was found, probably due to its low number of participants (18 patients) [23]. Thus, MDD treatment's response is decidedly a complex phenotype involving genetic and environmental factors.

Ketamine is an antidepressant drug that provides a fast, robust, and transient effect [70]. It also seems to rapidly reduce suicidal ideation in treatment-resistant MDD patients, making it an attractive therapy [70–72], even as some studies disagree with this effect [73, 74]. Advances in science have shown a connection between this fast-acting drug and the BDNF translation and signaling [75], confirming BDNF protein's importance as a possible mediator for new antidepressants.

Su et al. [29] characterized the ketamine dosage effect on Chinese treatment-resistant depressive patients of different BNDF Val66Met (rs6265) genotypes by dividing the research participants into three groups with no statistical genotypic difference (*p* = 0.41): placebo, 0.2 mg/kg dosage, and 0.5 mg/kg dosage. None of the BDNF genotypes predicted the response to ketamine when comparing participants with at least one polymorphic A (Met) allele with the homozygous GG (Val/Val) individuals (*p* = 0.55) [29] nor clear evidence of ketamine efficacy reduction in patients with polymorphic allele A (Met) [29], similar to the Hu et al. [76] study. Nevertheless, a ketamine dose-related effect was found, as measured by the HAM-D score, with the 0.5 mg/kg dosage being more effective in treatment-resistant participants, i.e., those with more severe MDD [29].

Transcranial direct current stimulation (tDCS) as an antidepressant mechanism might increase prefrontal cortex activity and improve depressive symptoms [77]. Brunoni et al. [19] did not find an association between BDNF rs6265 (Val66Met) polymorphism and clinical response to tDCS, comparable to the findings of other studies [19, 78]. Contrarily, Bocchio-Chiavetto et al. [79] found that GG (Val/Val) genotype correlated to depressive symptom improvement after tDCS treatment in 36 depressive patients.

In addition to pharmacological treatments and transcranial stimulation focused on MDD, cognitive therapies are indicated to patients with MDD to help with resilience—known as an individual's ability to deal with stress or trauma, for example [18]. Regarding BDNF Val66Met (rs6265) polymorphism, Peters et al. [18] noted that participants with an A (Met) allele presented higher resilience scores compared to those of the GG (Val/Val) genotype (*p* = 0.037). Furthermore, cognitive therapy improved resilience (*p* = 0.001) and reduced depressive effects (*p* = 0.001), depending on their sex (*p* = 0.008) and genetic susceptibility (*p* = 0.048) [18].

In short, genetic factors may influence the available antidepressant treatment efficiencies. For this reason, pharmacogenomic studies are necessary to increase the understanding of the disease and promote a better quality of life for MDD patients.

### 4.5. Quality and Limitation Assessment of the Selected Articles

With the complete human genome sequencing and the possibility of gene therapy, studies to understand how the genetic and environmental factors influence different diseases have gained space in the scientific community. Although GWAS's benefits and limitations are still being analyzed [80, 81], Chang et al. [82] related BDNF dysregulation, among other genes, to MDD when analyzing coexpression meta-analysis and DNA variant genome-wide association studies. Recently, other genome-wide association studies have identified BDNF associations with several behavioral and cognitive attributes, such as “worry”/anxiety [83].

Therefore, replication of these studies in different populations, due to heterogeneity, is necessary for genetic research generalization. Hence, we employed the Genetic RIsk Prediction Studies (GRIPS) guideline to assess the quality of the association studies chosen for this systematic review (see Table S2, in the Supplementary Material).

Composed of 25 items, our group decided to use only 20 GRIPS guideline items to appraise the selected studies' methods, results, and discussion. Of the 17 studies evaluated, 17.6% failed to meet at least 6 of the 20 evaluated items (adequacy % of less than 75%). The most common noncompliances were the lack of information about the study design, the setting, and the sampling at each stage. Moreover, 17.6% of the studies analyzed did not discuss the limitations found in the research development, which is essential for replicating a study. Despite this noncompliance, a good part of the evaluated studies strongly recommended replicating these analyses in different populations to increase the comprehension of the different genetic mechanisms involved in MDD development and its associated environmental factors. Much of this recommendation is due to heterogeneity and their small sample sizes, decreasing the study's power and limiting the generalization. Nevertheless, further studies must be cautious as larger sample sizes might lead to population stratification.

## 5. Final Considerations

Genes' participation in disease development may take many forms. The BDNF Val66Met (rs6265) genetic variant and its altered serum levels in MDD can influence Central Nervous System neurobiology, pharmacogenomics, and even environmental factors, e.g., epigenetic response to childhood adversities. Even so, and noted in all aspects, including genotypic frequency, there is no consensus among the BDNF Val66Met (rs6265) genetic variant studies.

Despite the BDNF (rs6265) GG (Val/Val) genotype being most frequent in some of the different populations studied, genetic and environmental heterogeneity, including culture, are one of the factors that may lead to this noncompliance between the results and the controversy on whether the BDNF (rs6265) genetic variant and its serum protein levels are associated or not with MDD.

Another factor is that BDNF polymorphism and its protein levels have been correlated to several other mental disorders, including schizophrenia and bipolar disorder [84–86], which would place BDNF as a biomarker for mental illness in general and explain some of the discrepancies. Studies analyzing polygenetic risk scores might help narrow its influence in MDD.

Given this and the other studies' recommendations, further research into the BDNF gene connection to MDD must be conducted in different populations and with a significant sample size to understand BDNF's role and different mechanisms in MDD etiology/pathology. This understanding increases the possibility of providing MDD patients a better quality of life and reducing MDD underdiagnosis, which causes great concern in public health.

## Figures and Tables

**(a):** 

Author	Title	Year	Country	Objective	Sample size	BDNF genetic variant	Genotypic frequency (Val/Val)	Laboratorial method	Results	*p* value (case vs. control)	Odds ratio
Peters et al. [18]	BDNF Val66Met polymorphism and resilience in major depressive disorder: the impact of cognitive psychotherapy	2020	Brazil	Investigate the BDNF Val66Met polymorphism effect on MDD patients' resilience scores and response to cognitive therapy.	*n* = 106 F = 83 (78.3%) M = 23 (21.7%)	rs6265	77.4% (*n* = 82)	TaqMan	The BDNF Val66Met polymorphism may be related to resilience in MDD patients.	0.214	—
Brunoni et al. [19]	Association of BDNF, HTR2A, TPH1, SLC6A4, and COMT polymorphisms with tDCS and escitalopram efficacy: ancillary analysis of a double-blind, placebo-controlled trial	2020	Brazil	Investigate whether the proposed SNPs correlate with neuroplasticity and if monoamine neurotransmitters activity is associated with the transcranial direct current stimulation (tDCS) effectiveness in MDD.	*n* = 195 F = 132 (67.7%) M = 63 (32.3%)	rs6265	65.1% (*n* = 127)	MassARRAY SNP genotyping	The A (Met) allele did not affect depression symptom improvement in any of the treatments: placebo (*p* = 0.84), tDCS (*p* = 0.48), nor escitalopram (*p* = 0.98).	0.78	—
Aldoghachi et al. [20]	Screening of brain-derived neurotrophic factor (BDNF) single nucleotide polymorphisms and plasma BDNF levels among Malaysian major depressive disorder patients	2019	Malaysia	Determine the association of three BDNF variants (rs6265, rs1048218, and rs1048220) in MDD patients	*n* = 300 F = 203 (67.7%) M = 97 (32.3%)	rs6265, rs1048218, rs1048220	24.3% (*n* = 73)	PCR and sequencing	The A (Met) allele increases the risk of developing MDD in the Malaysian population.	0.0075^∗^	1.95

**(b):** 

Author	Title	Year	Country	Objective	Sample size	BDNF genetic variant	Genotypic frequency (Val/Val)	Laboratorial method	Results	*p* value (case vs. control)	Odds ratio
Froud et al. [21]	The relationship between dietary quality, serum brain-derived neurotrophic factor (BDNF) level, and the Val66met polymorphism in predicting depression	2019	Australia	Investigate the complex relationship between food quality, BDNF protein serum levels, and depression. Moreover, evaluate whether the BNDF Val66Met polymorphism influences this relationship.	*n* = 187 F = 136 (72.7%) M = 51 (27.3%)	rs6265	66.9% (*n* = 81)	Sequencing	Both higher serum BDNF (*p* < 0.001) and lower dietary quality scores (*p* = 0.037) were significantly detected in depression.	0.864	—
Ai et al. [22]	Plasma brain-derived neurotrophic factor (BDNF) concentration and the BDNF Val66Met polymorphism in suicide: a prospective study in patients with depressive disorder	2019	China	Investigate the correlation between BDNF protein concentration and its (196C>A) polymorphism and SSRI response in a Chinese MDD population.	*n* = 125 F = 76 (60.8%) M = 49 (39.2%)	rs6265	24% (*n* = 30)	PCR-RFLP	BDNF protein concentrations were significantly lower in patients who attempted or suffered from suicidal ideation. BDNF might be beneficial as a biomarker in antidepressant treatment response.	0.194	—
Kitzlerová et al. [23]	Interactions among polymorphisms of susceptibility loci for Alzheimer's disease or depressive disorder	2018	Czech Republic	Examine the contribution between multiple functional polymorphism interactions in the risk of MDD and Alzheimer's.	*n* = 68 F = 53 (77.9%) M = 15 (22.1%)	rs6265	77.9% (*n* = 53)	PCR-RFLP, per Chou et al. [24]	The G/A (Val/Met) genotype frequency is significantly lower in MDD, indicating its protective effects. Furthermore, the combination of 2 to 5 different gene polymorphisms has a significant cumulative effect on the severity of MDD.	0.79	0.44 (G/A)

**(c):** 

Author	Title	Year	Country	Objective	Sample size	BNDF genetic variant	Genotypic frequency (Val/Val)	Laboratorial method	Results	*p* value (case vs. control)	Odds ratio
Bassi et al. [25]	Interaction between polymorphisms in SLC6A4 and *BDNF* on major depressive disorder in sample of the Argentinean population	2018	Argentina	Analyze a possible association between MDD and the HTR2A, BDNF, and APOE gene polymorphisms in an Argentine population sample, previously studied using two polymorphisms in SLC6A4.	*n* = 95 F = 74 (77.9%) M = 21 (22.1%)	rs6265	60% (*n* = 57)	PCR-RFLP	Patients with at least one BDNF (A), 5HTTVNTR (10), and 5HTTLPR (S) allele were at increased risk for developing depression.	0.58	0.74 (A/A)
Youssef et al. [16]	Association of *BDNF* Val66Met polymorphism and brain BDNF levels with major depression and suicide	2018	USA	Identify the BDNF interrelationship in suicide, MDD, and reported childhood adversities by examining BDNF polymorphism and its protein levels in the prefrontal cortex, ACC, and postmortem brainstem in MDD cases, suicidal and nonsuicidal.	*n* = 45 F = 10 (22.2%) M = 35 (77.8%)	rs6265	46.7% (*n* = 21)	PCR-RFLP	The A (Met) allele presence correlates with a lower BDNF protein level in MDD patients' ACC and caudal brainstem areas. Furthermore, lower BDNF protein levels were also associated with reported childhood adversities and death by suicide.	0.019^∗^	—
Caldieraro et al. [26]	Val66Met polymorphism association with serum BDNF and inflammatory biomarkers in major depression	2018	Brazil	Evaluate the BDNF Val66Met polymorphism association with its protein serum levels and inflammatory markers in depressed outpatients.	*n* = 73 F = 63 (86.3%) M = 10 (13.7%)	rs6265	75.3% (*n* = 55)	TaqMan-real-time PCR	The A (Met) allele presence is associated with higher BDNF protein levels and decreased serum inflammatory markers in depressed patients than the Val/Val genotype (*p* = 0.001).	0.051	—

**(d):** 

Author	Title	Year	Country	Objective	Sample size	BDNF genetic variant	Genotypic frequency (Val/Val)	Laboratorial method	Results	*p* value (case vs. control)	Odds ratio
Wang et al. [27]	Association of DNA methylation in BDNF with escitalopram treatment response in depressed Chinese Han patients	2018	China	Identify if BNDF DNA methylation can predict the antidepressant response.	*n* = 85 F = 57 (67%) M = 29 (33%)	rs6265, rs7103411, rs11030101, rs141850	24.71% (*n* = 21)	PCR-RFLP	BDNF gene DNA methylation's lowest mean correlated with an impaired antidepressant response.	0.225	—
Han et al. [28]	The effects of 5HTTLPR and BDNF Val66Met polymorphisms on neurostructural changes in major depressive disorder	2018	Republic da Korea	Investigate the 5HTTLPR and BDNF Val66Met genetic variants and their interactions' effect with the cortical volume and the white matter integrity in MDD.	*n* = 95 F = 76 (80%) M = 19 (20%)	rs6265	21.1% (*n* = 20)	According to Caldieraro et al. [26]; Wang et al. [27]; Han et al. [28].	The A (Met) allele affected the FA value differently in the right uncinate fasciculus—its value is decreased in the MDD participants and increased in healthy participants.	0.453	—
Su et al. [29]	Dose-related effects of adjunctive ketamine in Taiwanese patients with treatment-resistant depression	2017	China	Characterize ketamine dose-related antidepressant effects on treatment-resistant depression patients with predominately lower activity BDNF genotypes-GA(Val/Met) and AA (Met/Met).	*n* = 71 F = 53 (74.6%) M = 18 (25.4%)	rs6265	17% (*n* = 12)	PCR and RFLP	Reduced ketamine efficacy is unclear in patients with the A (Met) allele.	0.41	—

**(e):** 

Author	Title	Year	Country	Objective	Sample size	BNDF genetic variant	Genotypic frequency (Val/Val)	Laboratorial method	Results	*p*-value (case vs. control)	Odds ratio
Tatham et al. [30]	The *5HTTLPR* and *BDNF* polymorphisms moderate the association between uncinate fasciculus connectivity and antidepressants treatment response in major depression	2017	Canada	Assess if the white matter integrity indices and the association between 5HTTLPR and BDNF (val66met) polymorphisms predict the magnitude of change in depressive symptoms after antidepressant treatments.	*n* = 46 F = 26 (56.5%) M = 20 (43.5%)	rs6265	80.44% (*n* = 37)	PCR-RFLP	A (Met) allele carriers exhibited a reduction in MDD improvement; furthermore, BDNF gene polymorphisms significantly impacted the uncinate fascicle's FA values.	0.13	—
Tatham et al. [17]	White matter integrity in major depressive disorder: implications of childhood trauma, *5HTTLPR* and *BDNF* polymorphisms	2016	Canada	Evaluate how childhood neglect and the 5HTTLPR and BDNF polymorphisms influence the brain's myelin integrity in MDD.	*n* = 55	rs6265	80% (*n* = 42)	PCR-RFLP	A (Met) patients have reduced FA values in the nonisolated fascicle compared with GG (Val/Val) patients (*p* = 0.005). BDNF polymorphism moderated the association between the depression severity and FA levels in the uncinate fascicle (*p* = 0.02).	0.197	—
Jaworska et al. [31]	The influence o*f 5HTTLPR* and Val66Met polymorphism on cortical thickness and volume in limbic and paralimbic regions in depression: a preliminary study	2016	Canada	Evaluate the 5HTTLPR and BDNF gene polymorphism influence on cortical thickness or brain volume, in the paralimbic and limbic regions, in the MDD.	*n* = 43 F = 26 (60.5%) M = 17 (39.5%)	rs6265	81.3% (*n* = 35)	PCR and RFLP	BNDF polymorphism did not affect the paralimbic and limbic structures' cortical thickness or cerebral volume.	>0.05	—

**(f):** 

Author	Title	Year	Country	Objective	Sample size	BDNF genetic variant	Genotypic frequency (Val/Val)	Laboratorial method	Results	*p* value (case vs. control)	Odds ratio
Kostic et al. [32]	The cumulative effect of genetic polymorphisms on depression and brain structural integrity	2016	Serbia	Evaluate the SERT, BDNF, COMT gene functional polymorphisms accumulation effect on specific MDD patients' brain structures.	*n* = 77 F = 46 (60%) M = 31 (40%)	rs6265	69% (*n* = 53)	PCR-RFLP	High probability of G (Val) (BDNF), Met (COMT), and L (SERT) alleles being present in MDD patients compared to control.	<0.001	—
Cao et al. [33]	Reduced hippocampus volume and memory performance in bipolar disorder patients carrying the BDNF val66met met allele	2016	USA	Investigate the BDNF gene polymorphism effect on hippocampal volume and memory performance in adults diagnosed with bipolar disorder type I and MDD.	*n* = 33 F = 23 (69.7%) M = 10 (30.3%)	rs6265	75.8% (*n* = 25)	TaqMan	MDD patients and A (Met) allele carriers have similarities in the hippocampus volume and cognitive performance compared with the control group.	0.72	—

^∗^ *p* value = <0.05. MDD = major depressive disorder; Val (valine) = G allele; Met (methionine) = allele A; FA = fractional anisotropy; ACC = anterior cingulate cortex; tDCS = transcranial direct current stimulation; *n* = number; F = female; M = male.

## Data Availability

Data used in the review are those from the article's references. The articles that passed the inclusion criteria were analyzed according to the Genetic RIsk Prediction Studies (GRIPS) guideline to determine their quality. We only used other references to discuss or clarify points from the selected articles presented in Table 1.

## Supplementary Materials

Supplementary Materials Table S1: articles not selected following the PECOS strategy and the eligibility criteria. Table S2: evaluation of the articles' quality, according to the adapted GRIPS guideline.
