# Supplementary material for: BDNF Genetic Variant and Its Genotypic Fluctuation in Major Depressive Disorder
Source: Behav Neurol. 2021 Nov 1;2021:7117613. doi: 10.1155/2021/7117613 (PMC8575598; doi:10.1155/2021/7117613)
Supplement: Supplementary Materials — Table S1: articles not selected following the PECOS strategy and the eligibility criteria. Table S2: evaluation of the articles' quality, according to the adapted GRIPS guideline. [file 7117613.f1.zip › Table S1 - BDNF genetic variant and MDD.pdf]

Table S1: Articles not selected following the PECOS strategy and the eligibility criteria.

Author	Title	Year	Exclusion Phase	Reason for exclusion
Lye et al.	Predictors of recurrence of major depressive disorder.	2020	Complete text	Incomplete data (frequency)
Schosser et al.	The Impact of BDNF Polymorphisms on Suicidality in Treatment-Resistant Major Depressive Disorder: A European Multicenter Study.	2018	Complete text	Incomplete data (frequency)
Van Venzel et al.	Effect of childhood maltreatment and brain-derived neurotrophic factor on brain morphology.	2016	Complete text	Incomplete data (frequency)
Losenkov et al.	Association Between BDNF Gene Variant Rs6265 and the Severity of Depression in Antidepressant Treatment-Free Depressed Patients	2020	Complete text	Incomplete data (frequency)
Hsieh et al.	Abnormal Brain-Derived Neurotrophic Factor Exon IX Promoter Methylation, Protein, and mRNA Levels in Patients with Major Depressive Disorder	2019	Complete text	Incomplete data (frequency)
Oh et al.	The Role of Dendritic Brain-Derived Neurotrophic Factor Transcripts on Altered Inhibitory Circuitry in Depression	2019	Complete text	Incomplete data (frequency)
Calabro et al.	Neuroplasticity, Neurotransmission and Brain-Related Genes in Major Depression and Bipolar Disorder: Focus on Treatment Outcomes in an Asiatic Sample	2018	Complete text	Incomplete data (frequency)
Sun et al.	GSK-3 beta and BDNF genes may not be associated with venlafaxine treatment response in Chinese of Han ethnicity	2019	Complete text	Incomplete data (frequency)

Author	Title	Year	Exclusion Phase	Reason for exclusion
Schoroeter et al.	Longitudinal multi-level biomarker analysis of BDNF in major depression and bipolar disorder.	2020	Complete text	Incomplete data (frequency)
Sun et al.	Effects of polymorphisms of serotonin transporter promoter (5-HTTLPR) and brain derived neurotrophic factor gene (G196A rs6265) on the risk of major depressive disorder in the Chinese Han population.	2016	Complete text	Incomplete data (frequency)
Kao et al.	Gene-based analysis of genes related to neurotrophic pathway suggests association of BDNF and VEGFA with antidepressant treatment-response in depressed patients	2018	Complete text	Wrong Gene
Rahmani et el.	The Brain-Derived Neurotrophic Factor: Missing Link Between Sleep Deprivation, Insomnia, and Depression.	2020	Complete text	Review/Meta-analysis
Ferrer et al.	BDNF genetic variants and methylation: effects on cognition in major depressive disorder.	2019	Title and abstract	Wrong Gene
Deflesselle et al.	The TRKB rs2289656 genetic polymorphism is associated with acute suicide attempts in depressed patients: A transversal case control study	2018	Title and abstract	Wrong Gene
Bondarenko et al.	Genetic Analysis of BDNF, GNB3, MTHFR, ACE and APOE Variants in Major and Recurrent Depressive Disorders in Russia.	2016	Title and abstract	Wrong Gene
Maul et al.	Genetics of resilience: Implications from genome-wide association studies and candidate genes of the stress response system in posttraumatic stress disorder and depression	2020	Title and abstract	Wrong Gene
Klucken et al.	Failure to Replicate the Association Between Fractional Anisotropy and the Serotonin Transporter Gene (5-HTTLPR, rs25531)	2018	Title and abstract	Wrong Gene

Author	Title	Year	Exclusion Phase	Reason for exclusion
Kautzky et al.	Epistasis of HTR1A and BDNF risk genes alters cortical 5-HT1A receptor binding: PET results link genotype to molecular phenotype in depression.	2019	Title and abstract	Wrong population
Amadio et al.	BDNFVal66met polymorphism: a potential bridge between depression and thrombosis.	2017	Title and abstract	Wrong population
Prats et al.	Neurotrophins role in depressive symptoms and executive function performance: Association analysis of NRN1 gene and its interaction with BDNF gene in a non-clinical sample.	2017	Title and abstract	Wrong population
Antypa et al.	Clinical and genetic factors associated with suicide in mood disorder patients	2016	Title and abstract	Wrong population
Stephani et al.	Polymorphism of the brain-derived neurotrophic factor and dynamics of the seizure threshold of electroconvulsive therapy.	2017	Title and abstract	Wrong population
Osorio et al.	Adapting to Stress: Understanding the Neurobiology of Resilience	2017	Title and abstract	Wrong population
Sigitova et al.	Biological hypotheses and biomarkers of bipolar disorder	2017	Title and abstract	Wrong population
Maron et al.	Biological markers of generalized anxiety disorder	2017	Title and abstract	Wrong population
Wheeler et al.	BDNF-Dependent Effects on Amygdala-Cortical Circuitry and Depression Risk in Children and Youth.	2018	Title and abstract	Wrong population
Vinberg	Risk. Impact of having a first-degree relative with affective disorder: a 7-year follow-up study	2016	Title and abstract	Wrong population
Meyer et al.	A genetic variant brain-derived neurotrophic factor (BDNF) polymorphism interacts with hostile parenting to predict error-related brain activity and thereby risk for internalizing disorders in children	2018	Title and abstract	Wrong population

Author	Title	Year	Exclusion Phase	Reason for exclusion
Udina et al.	Glucocorticoid Receptors, Brain-Derived Neurotrophic Factor, Serotonin and Dopamine Neurotransmission are Associated with Interferon-Induced Depression.	2016	Title and abstract	Wrong population
Wang et al.	The association between BDNF Val66Met polymorphism and emotional symptoms after mild traumatic brain injury.	2018	Title and abstract	Wrong population
Lundwall et al.	Genetic associations with reflexive visual attention in infancy and childhood	2017	Title and abstract	Wrong population
Van Den et al.	Hippocampal volume change following ECT is mediated by rs699947 in the promotor region of VEGF	2019	Title and abstract	Wrong population
Bogdan et al.	Genetic Moderation of Stress Effects on Corticolimbic Circuitry	2016	Title and abstract	Wrong population
Notaras et al.	Neurobiology of BDNF in fear memory, sensitivity to stress, and stress-related disorders.	2020	Title and abstract	Review/Meta-analysis
Dincheva et al.	The Role of BDNF in the Development of Fear Learning.	2016	Title and abstract	Review/Meta-analysis
Douglas et al.	High-resolution chromosome ideogram representation of recognized genes for bipolar disorder.	2016	Title and abstract	Review/Meta-analysis
Badamasi et al.	Genetic endophenotypes for insomnia of major depressive disorder and treatment-induced insomnia.	2019	Title and abstract	Review/Meta-analysis
Hoppen et al.	Childhood adversity as a transdiagnostic risk factor for affective disorders in adulthood: A systematic review focusing on biopsychosocial moderating and mediating variables	2018	Title and abstract	Review/Meta-analysis
Pereira et al.	Imaging genetics paradigms in depression research: Systematic review and meta-analysis	2018	Title and abstract	Review/Meta-analysis
Amare et al.	Pharmacogenomics in the treatment of mood disorders: Strategies and Opportunities for personalized psychiatry	2017	Title and abstract	Review/Meta-analysis

Author	Title	Year	Exclusion Phase	Reason for exclusion
Fabbri et al.	Consensus paper of the WFSBP Task Force on Genetics: Genetics, epigenetics and gene expression markers of major depressive disorder and antidepressant response	2017	Title and abstract	Review/Meta-analysis
Zhang et al.	Neuroimaging genomic studies in major depressive disorder: A systematic review	2018	Title and abstract	Review/Meta-analysis
Gottschalk et al.	Genetics of generalized anxiety disorder and related traits	2017	Title and abstract	Review/Meta-analysis
Davydova et al.	Genetic basis of depressive disorders	2019	Title and abstract	Review/Meta-analysis
Savage et al.	The Genetics of Anxiety-Related Negative Valence System Traits	2017	Title and abstract	Review/Meta-analysis
Pereira et al.	The relationship between genetic risk variants with brain structure and function in bipolar disorder: A systematic review of genetic-neuroimaging studies	2017	Title and abstract	Review/Meta-analysis
Smeeth et al.	Polygenic risk for circulating reproductive hormone levels and their influence on hippocampal volume and depression susceptibility	2019	Title and abstract	Review/Meta-analysis
Gonda et al.	Genetic variants in major depressive disorder: From pathophysiology to therapy.	2019	Title and abstract	Review/Meta-analysis
Busch e Menke	Blood-based biomarkers predicting response to antidepressants	2019	Title and abstract	Review/Meta-analysis
Hing et al.	A comprehensive review of genetic and epigenetic mechanisms that regulate BDNF expression and function with relevance to major depressive disorder.	2018	Title and abstract	Review/Meta-analysis
